# Masculinity as a barrier to men's use of HIV services in Zimbabwe

**DOI:** 10.1186/1744-8603-7-13

**Published:** 2011-05-15

**Authors:** Morten Skovdal, Catherine Campbell, Claudius Madanhire, Zivai Mupambireyi, Constance Nyamukapa, Simon Gregson

**Affiliations:** 1 Institute of Social Psychology, London School of Economics and Political Science, London, UK; 2 Biomedical Research and Training Institute, Harare, Zimbabwe; 3 Department of Infectious Disease and Epidemiology, Imperial College, London, UK

**Keywords:** Gender, masculinity, ART access, VCT, AIDS, HIV services, Africa

## Abstract

**Background:**

A growing number of studies highlight men's disinclination to make use of HIV services. This suggests there are factors that prevent men from engaging with health services and an urgent need to unpack the forms of sociality that determine men's acceptance or rejection of HIV services.

**Methods:**

Drawing on the perspectives of 53 antiretroviral drug users and 25 healthcare providers, we examine qualitatively how local constructions of masculinity in rural Zimbabwe impact on men's use of HIV services.

**Results:**

Informants reported a clear and hegemonic notion of masculinity that required men to be and act in control, to have know-how, be strong, resilient, disease free, highly sexual and economically productive. However, such traits were in direct conflict with the 'good patient' persona who is expected to accept being HIV positive, take instructions from nurses and engage in health-enabling behaviours such as attending regular hospital visits and refraining from alcohol and unprotected extra-marital sex. This conflict between local understandings of manhood and biopolitical representations of 'a good patient' can provide a possible explanation to why so many men do not make use of HIV services in Zimbabwe. However, once men had been counselled and had the opportunity to reflect upon the impact of ART on their productivity and social value, it was possible for some to construct new and more ART-friendly versions of masculinity.

**Conclusion:**

We urge HIV service providers to consider the obstacles that prevent many men from accessing their services and argue for community-based and driven initiatives that facilitate safe and supportive social spaces for men to openly discuss social constructions of masculinity as well as renegotiate more health-enabling masculinities.

## Introduction

The World Health Organisation [1] states that gender differences must be acknowledged and addressed if HIV and AIDS programmes are to be effective. Differences in HIV service^i ^uptake have been identified, with a growing number of studies highlighting that men are significantly less likely to get tested for HIV [2-4] or to enroll and adhere to antiretroviral treatment (ART) services [5-8]. In South Africa, for example, a survey found that only one out of five people tested for HIV were male [3] and an investigation into HIV testing in a multi-country HIV workplace programme in sub-Saharan Africa found that male workers (22%) compared to female workers (28%) and male spouses (6%) compared to female spouses (18%), were less likely to take advantage of the programme and get tested for HIV [9]. Even where an equal proportion of men and women are found to make use of HIV testing services, men are observed to only get tested for HIV after becoming severely ill [4]. It follows from men's relatively poor and delayed uptake of HIV testing services that in many contexts women outnumber men in accessing ART. Estimates of ART enrollment in low- and middle-income countries suggest that at the end of 2008, 45% of women and 37% of men who qualify for treatment were enrolled onto an HIV care and treatment programme [10]. This trend is supported by a systematic review of 21 peer-reviewed articles and reports describing the gender distribution of patients accessing ART in sub-Saharan Africa. This review found that, in the majority of studies, 60% more women enrolled onto ART compared to their male counterparts - a trend which they argue is not explained by the higher HIV prevalence amongst women compared to men, but an indicator of gendered health seeking behaviours [7]. Also adherence to ART is highly gendered. A study in South Africa found that 70% of those who managed to stay on and adhere to HIV treatment were women [5] and in Uganda, just over twice as many women managed to keep viral suppression high after six months of treatment [8]. These differences, coupled with the fact that men get infected at an older age and thereby experience disease progression faster than women, may help us understand why the rate of AIDS-related mortality in men is higher than in women in many places in Africa [11,12], underlining the need to explore the causes of men's relative disadvantage. Against this background there is an urgent need to sketch out the pathways through which gendered constructions impact on men's use of HIV testing services, uptake of ART and adherence to antiretroviral drugs.

It is clear from the published work that men face particular challenges in accessing and adhering to HIV treatments. Most studies examining the gender distribution of HIV services tend to be quantitative, so offer little insight to the social processes that contribute to men's relative disadvantage in HIV service uptake and retention [13]. However, a couple of recent studies point towards social constructions of masculinity as one possible explanation for men's poor uptake and participation in HIV services. Izugbara, Undie et al. [14] found that young male adolescents in Malawi and Uganda resisted getting tested for HIV as they felt it signalled lack of self-confidence and an acknowledgement of their vulnerability, traits that conflicted with their male youth identity. Similarly, Fitzgerald, Collumbien and Hosegood [15] have highlighted some of the specific challenges facing South African men in participating in antiretroviral treatment programmes, arguing that gendered expectations and local constructions of masculinity discouraged some men from disclosing their HIV status and seeking treatment in fear that they would be perceived as failing sons, husbands or breadwinners. Coping with the stresses of HIV and treatment, many men resorted to alcohol, further undermining their treatment regimens (ibid.). The problem of 'male-unfriendly' services is not confined to the HIV/AIDS field. In their critique of international health services research, Lee and Owens [16] identify a general tendency to neglect the socio-cultural and geo-political influences on men's health-related behaviours, arguing that such a neglect often implies that men's poor health-service uptake is down to individual choice. It is against this background, and in our interest to advance HIV services for men, that we explore how masculinity serves as a barrier to men's uptake of HIV testing and treatment services in Zimbabwe and highlight the pathways through which some men manage to resist hegemonic masculinities and enroll onto ART.

## Hegemonic masculinity and HIV/AIDS

Connell's [17] theory of hegemonic masculinity, and Morrell's discussions of how hegemonic masculinities are framed by the complex dynamics of race and class inequalities in southern Africa [18], form the backdrop to our interest in differences in health-seeking behaviours of men and women in the context of HIV/AIDS. Connell [17] defines hegemonic masculinity as the enactment of an idealised form of masculinity (being 'the real man') in a particular time and place. Whilst hegemonic masculinity is often seen as a process that is both subordinating of women, as well as other forms of masculinities (such as those exhibited by homosexuals), we argue in this paper that men's enactment of social constructed versions of manhood can also have a subordinating role, by preventing them from taking advantage of life-saving HIV services [19]. This resonates with Clatterbaugh's [20] argument that, as men participate in the construction of powerful masculinities and patriarchies, they often place themselves in a disadvantaged position when it comes to health care access. Views of men and their behaviours associated with hegemonic masculinities can therefore be deeply restrictive to men [16].

Needless to say, dominant forms of masculinity more often than not work to men's advantage in all sorts of ways, particularly in relation to their privileged access to power and influence in the socio-economic and political spheres, and also often in the private sphere. Just to cite one example of this complexity, Campbell [19,21] tracks the complex mix of ways in which migrant labour and poverty shape masculinities of southern African migrant mineworkers in ways that both promote and undermine men's well-being. Thus, for example, her study highlights the way in which hegemonic masculinities promote male survival in dangerous and difficult work conditions in mines in South Africa - yet at the same time, and in line with the argument of this paper, place men at greater risk of HIV as they spend their evenings away from home with different sexual partners.

As the pathways through which hegemonic masculinities impact on HIV service uptake and ART adherence are complex and multi-faceted, we use two inter-linked conceptual tools alongside Connell's notion of hegemonic masculinity to frame our own findings. Social representations theory enables us to view hegemonic and other masculinities as continually in the making, providing a theoretical space for us to consider ways in which masculinities might be contested and transformed in specific settings. The notion of therapeutic citizenship enables us to highlight ways in which characteristics of hegemonic masculinity may stand in a problematic relationship with the practical requirements of health service and drug treatment settings. Each is discussed below.

### Social representations of masculinity

The first theory informing our work is social representations theory (SRT). Social representations are forms of knowledge that are socially constructed, including values, ideas and practices, which enable people to orientate themselves in their social world [22]. These include local constructions of gender and gendered identities. In the process of identifying themselves as men, individual men will have to situate themselves in relation to the norms and representations that define dominant notions of masculinity in particular contexts [23-25]. SRT views socially constructed knowledge systems and identities as dynamic, rather than static, and capable of transformation through interaction between people, groups and organisations (ibid.). This suggests that, under the right conditions and provided with opportunities, men can renegotiate and critically engage with social representations of what constitutes a 'real man' in a particular context.

Studies in South Africa [25] have alluded to some of the traits that men in certain cultural contexts are expected to possess. These include being tough, unemotional, aggressive, denying weakness, sexually unstoppable, appearing physically strong and in competition with other men. However, as Courtenay [26] highlights, men are not only conditioned and socialised by social representations of manhood, they are also active agents in constructing and enacting these representations in their own lives. Disengagement with health services and carelessness of health and well-being may be one way to demonstrate hegemonic masculinity [26]. Furthermore, health-risk behaviours, such as having unprotected sex and multiple sexual partners, may be directly associated with virility and therefore a way to assert their manliness in society [23-25,27]. Taking this to the extreme, men in a particular context in Malawi were found to speak about HIV as something of a symbol of their manhood [28]. But in what ways do social representations and local constructions of masculinity interact and conflict with AIDS treatment?

### Gendering of therapeutic citizenship

The concept of therapeutic citizenship provides a useful frame for our investigation of the interface between masculinity and AIDS treatment. Developed through work in West Africa, Nguyen and colleagues [29,30] report on the socio-cultural and historical context of ART access and adherence in Africa. They define therapeutic citizenship in terms of the identities and associated practices that ART patients need to adopt in order to gain access to the very limited supply of HIV services available to them [29,30]. This framework allows us to move beyond a narrow focus on locally circumscribed cultural contexts of treatment, widening our lens to take account of global assemblages of organisations, norms and practices which constitute the global public health framework within which ART treatment is conceptualised and provided, and, more specifically, the interface between this global assemblage and HIV services in local settings. Nguyen defines therapeutic citizenship as "a biopolitical citizenship, a system of claims and ethical projects that arise out of the conjugation of techniques used to govern populations and manage individual bodies", which may not always fit well with the local identities of those who at the receiving ends of biomedical treatment regimes [[29]: p.126]. Nguyen argues for an understanding of HIV service provision that views even local settings as the culmination of a hybrid of discourses and practices of biomedics, policy makers and service providers world-wide.

In this vein, Richey [31] highlights how the requirements of therapeutic citizenship may conflict with social expectations and social identities at a local level. In her study on ART and reproductive health services in South Africa, Richey found women of reproductive age and on ART had to negotiate between being good therapeutic citizens (global expectation) and fulfilling their social position as sexual beings with a wish and role to procreate (local expectation). She argues that more attention needs to be given to the gendering of biomedical interventions such as ART, something we seek to do in this paper in relation to masculinity. To do this we explore *how *ART may conflict with local versions of manhood. A study and review commissioned by the World Bank highlights a common perception among men in sub-Saharan Africa that clinics are 'female spaces' and that a real man does not fall ill [32]. It is clear that such perceptions may prevent some men from accessing services. Acknowledging this dilemma, Colvin and Robins [33], through their work with South African support groups for men with HIV, highlight how support groups can provide men with a social space to negotiate questions of masculinity and identity, enabling them to develop new kinds of masculinities that are more aligned to the therapeutic citizenship that is required for them to access and adhere to ART. To understand the processes that prevent men from accessing HIV services and the ways in which men negotiate new masculinities in order to access and adhere to ART, we seek to expand on this early work by explicitly exploring the role of hegemonic masculinities in ART provision.

In summary, men's health beliefs and behaviours are both conditioned by socially constructed expectations of what it means to be a man *and *are deliberately enacted by men to demonstrate their 'manhood', taking an active role in constructing dominant norms of masculinity. This, coupled with the expectations of how an AIDS patient should behave in order to access biomedical services, highlights the importance of mapping out the ways in which masculinity is socially negotiated and transformed and continues to limit many men's uptake of HIV services.

## Methodology

We report on a qualitative case study to answer the following research question: What factors limit the uptake of HIV services by men in rural Zimbabwe? We address this question using data from a larger study of HIV services in Zimbabwe. Ethical permission to conduct this study was granted by the Medical Research Council of Zimbabwe (A/681) and Imperial College London (ICREC_9_3_13). Informed and written consent was gathered from all research participants with the agreement that their identities would not be revealed. Pseudonyms have been used throughout.

### Study area and sampling

We conducted the study in the Manicaland province of eastern Zimbabwe where the HIV rate is estimated at 18% [34]. Informants were recruited from health centres in three sub-locations of Manicaland. The areas are characterised by poverty and residents in this area are primarily subsistence farmers living in rural homesteads, often without electricity or plumbing. Many of those in formal employment (predominantly men) tend to work in the cities and send money back to their rural homes. Many families, particularly those affected by AIDS, rely on food aid from international organizations and the goodwill of church and community support groups. The health centres in our study areas have experienced rapid growth in the number of people accessing ART and deal with frequent staff, equipment and electricity shortages.

In mapping out the social representations of masculinity that limit men's uptake of services, we draw on a study of the user-service interface, involving 78 participants - including male and female ARV users (n = 53) and health staff (n = 25) (see Table 1). We recruited ART patients/carer participants through openly HIV-positive community members and from hospital or clinic sites and discussed the struggles they faced before accessing HIV services. Informants were approached in hospital and clinic waiting rooms and invited to participate. Researchers' requests to interview a person on ART were only refused in one case, where the person said he did not have time to participate. Health staff interviewed included primarily nurses but also included HIV counsellors, pharmacists and a clerk. Permission to conduct the research in or around the health facility was granted by each health centre's doctor or nurse-in-charge. Staff members were subsequently approached individually and asked if they were willing to participate; all agreed.

**Table 1:** Summary of participants and research methods

	Interviews	*Male*	*Female*	FGD	*Male*	*Female*	Participants	*Male*	*Female*
Health staff	**18**	9	9	**1**	5	2	**25**	14	11
ARV users	**19**	13	6	**4**	8	26	**53**	21	32
Total	**37**	22	15	**5**	13	28	**78**	35	43

A limitation of this study is that it does not include those men who may believe that they are HIV positive, but decide not to make use of HIV services. Notwithstanding this limitation, our research interviews contained a wealth of information about the impacts of masculinity on service uptake, from both nurses and patients, with the latter including several men who had initially been reluctant to get tested and treated.

### Data collection and analysis

This study was part of a wider research project into the service-user interface in the context of ART in Zimbabwe [35-40]. We conducted 19 individual and 4 group interviews with adult ARV users, 21 individual and 3 group interviews with carers for children on ART, and 18 individual and one group interview with health staff (see Table 1). Topic guides explored issues surrounding HIV testing, ART uptake and adherence, disclosure of HIV and experiences at the health care centre. To gather more specific information about differences and experiences in HIV service access, participants were asked: 'What factors do you think have the most impact on access?', 'Why do some patients fail to present themselves at services?', 'Can you give me an example of a person with HIV who was accessed the services appropriately?' and 'Can you give me an example of a patient who failed to access the services at the best time?' Most respondents volunteered information about gender differences in response to these open-ended questions, and the interviewers used probing questions to gather more detail. Interviewers had been alerted to our interest in gender, so made a special point of probing any references made to this topic. During focus groups with patients, participants were invited to perform a role play of 'a good visit to the health centre' and 'a bad visit to the health centre'. These role-plays revealed a great deal about how HIV services users perceive the friendliness and accessibility of HIV services for both men and women.

The interviews were conducted by three experienced Shona-speaking fieldworkers who, with permission from the informants, audio-recorded the interviews. All audio files were translated into English and transcribed by the fieldworkers. To thank the informants, FGD participants were given soap, and interviewees were given a T-shirt. All transcripts were imported into Atlas.Ti, a qualitative analysis software package, through which we began coding the data set. For the aims of this paper, we repeatedly read and re-read the interview transcripts for any information relating to factors shaping men's uptake to services. Using Attride-Stirling's thematic network analysis, we identified 41 codes, covering 26 basic themes which were further clustered into 9 organising themes, and 3 global themes (Table 2). These global and organising themes form the structure of our findings section.

**Table 2:** Thematic Network (from codes to global themes)

Codes	Basic themes identified	Organising themes	Global themes
- Men feel superior Strong and resilient - Independent and tough - Pride - Can't show fear	1. Men are perceived as physically strong and capable of withstanding disease. 2. Men are perceived as emotionally independent and tough. 3. Men should not show fear.	Characteristics of 'a real man'	**Social constructions of masculinity**
- Gender roles - Men are head of house - Changes in gender roles	4. Men are perceived as breadwinners and the ones to carry out heavy duties, whilst women work at home, providing care for children and support husbands. 5. As households get affected by AIDS, gender roles get more fluid.	Men's roles and responsibilities	
- Men have girlfriends - Women not allowed extra-marital relationships - Men's sexual desires need to be met - Men bond in beer halls	6. Unlike women, it is common and a virtue for men to have multiple sexual partners. 7. Men are perceived to have sexual urges that need to be met. 8. Beer halls are a common space for men to meet girlfriends and assert their manhood to other men.	Sexuality and manhood	

- Fear of being recognised as HIV positive - Afraid of being stigmatised - Having HIV/AIDS exposes their promiscuity - Embarrassment - Fear disclosing status to their wives - Fear being alone - HIV compromises their manhood	9. Whilst having multiple sexual partners is a sign of virility, many men reported feeling embarrassed from failing to protect themselves. 10. Healthy sexuality is linked to a hegemonic masculine sexuality.11 Men fear loosing their dignity and being stigmatised. 12. Married men fear being abandoned by their wives and young men fear being rejected by girls and living a life alone.	Men's fear of HIV	**Barriers to men's HIV services uptake**
- Not taking HIV/AIDS seriously - Avoiding to talk about AIDS - Fatalism and risk taking - Denying it can happen to them - Death over dishonour - Passing on the blame - Drink alcohol to avoid reality - Making excuses to avoid getting tested for HIV	13. Few men want to acknowledge the seriousness of AIDS and avoid talking about it. 14. Many men do not believe it can happen to them, but take risks as accidents are unavoidable. 15. Men drink, blame others and ignore health services in order to 'avoid' the reality of AIDS.	Delusion, denial and diversion	
- Hospitals are female spaces - Men struggle to adhere to ART - Male behaviours conflict with treatment schedules	17. Hospitals are seen as spaces for women and children, not for men. 18. Elements of the ART treatment regimen conflicts with male behaviours that define their manhood.	Masculinity conflicts with 'patient persona'	

- Wives encourage husbands - Men seek treatment, but delayed - Men are brought in wheel barrows to the hospital	19. Couple testing and men getting support from their wives encourage men to seek HIV testing. 20. Men delay seeking treatment but are eventually brought to hospital in wheel barrows and treatment commences.	Persuasion and need	**Facilitators of men's usage of HIV services**
- Men lack information, so benefit from counselling - Reaching men through interventions - Food aid	21. Men often lack knowledge about HIV and treatment services and benefit tremendously from receiving counselling. 22. Food aid given to ART users can encourage men to seek HIV testing/ART as it will help them fulfil their role as breadwinners. 23. Support groups provide men with an opportunity to renegotiate their masculinities.	Gender sensitive HIV management services	
- Acceptance of HIV status - Change and reflection - Us and them - Challenge stigma	24. HIV positive men on ART break away from hegemonic masculinities. 25. HIV positive men on ART see themselves as responsible and valuable citizens. 26. HIV positive men on ART seek to resist stereotypes.	Constructing responsible masculinities	

## Findings

Whilst we did not interview men who had failed to take up services ourselves, our data set included a lot of talk about men and services and a general consensus, echoing findings from elsewhere in sub-Saharan Africa, that fewer men than women made use of HIV services and often only when seriously ill.

"Even in our cohorts, we have very few men. We can initiate a cohort of 30 patients, you will find only one male and the other 29 will be women." Claudius, nurse

Our informants' accounts of men's journey from realising they may have HIV and fearing the consequences of it, to having come to terms with their status and enrolled onto an ART programme, converged on a small number of common themes which are detailed in our thematic analysis.

### Social constructions of masculinity

A clear representation of hegemonic masculinity dominated people's accounts of their social reality and their explanations of why men did not take advantage of HIV services. Whilst it was clear that not all men subscribed to this notion of hegemonic masculinity, and others had managed to resist it in making the decision to be tested and treated, it served as a very clear and identifiable reference point in all the interviews. People engaged in constant debate with this notion of masculinity, taking up different positions in relation to it, but always using it as a yardstick against which they defined themselves.

#### Characteristics of 'a real man'

Men's experiences of self and other were constructed around their accounts of those 'manly' characteristics which distinguished them as superior from their 'weaker' female counterpart. Men perceived themselves as physically strong, tough, resilient, problem solvers and capable of withstanding 'little illnesses'.

"Men are just stronger in terms of resilience... as men, we have been given toughness such that we can pull through even the most difficult situation." James, patient

Women spoke of men differently and often with an awareness of the kind of pressure men were under to be 'a real man'. As Marta, a nurse, also points out, men do not want to show fear. Men are not supposed to show emotion or anxiety about their own welfare. On the contrary, showing they do not care is an essential way in which they 'perform' or construct themselves as men.

"Men, as I see them, don't want to know about their status when they are fit and strong, they do not want to appear afraid I think. Not wanting to know what their status is, is like saying 'I'm strong I'm strong'." Marta, nurse

Charles comments that such expectations make it particularly hard to be a man.

"I feel being a man is really hard in this community. Once you finish school, parents will not help with anything if you are a man. It is different with ladies who still receive the support of the family even when they finish school. Men are expected to be hard enough and strong enough to look after themselves. So I feel the community expect a lot of toughness from us men." Charles, patient

Charles, a man who had managed to access and benefit from services, shows some insight into the socially constructed nature of masculinity in this context. In our study, in accounting for their own acceptance of their HIV status and need for help, male patients often described themselves as different from other men in this regard.

#### Men's roles and responsibilities

Being 'a real man' translated into many roles and responsibilities. Men, for example, were perceived as household providers and the ones to carry out responsibilities requiring physical strength, whilst women do household duties such as cooking, cleaning and caring for children.

"In terms of household chores, men are supposed to do all the manly duties like looking after livestock and doing most of the farming, while women concentrate with things like fetching water, washing and cooking for the whole family. In this community it is generally men who are supposed to make sure they provide the financial needs of the family, so this includes paying school fees for children." Emmanuel, patient

Such perceptions pressure men into believing that those who cannot fulfill their roles and responsibilities as breadwinners and heads-of-house are not 'real' men, fathers or husbands. It is precisely such perceptions that add to the stress of manhood, making it difficult for men to behave in counter-stereotypical ways. However, as Charles indicated earlier, male ART patients who have come to terms with their HIV status and the physical limitations that come with the disease, adopt a different and more reflective masculinity. Carl, another patient who has constructed a non-hegemonic account of his own masculinity, indicates that, in this family, his AIDS has encouraged them to adopt more fluid gender roles and he gladly engages in cooking and child minding.

"People say that cooking and child care are duties of the wife and any man who does such kind of duties is either bewitched by the wife or is just weak (he laughs). But I don't see anything wrong in cooking and looking after the children when I am at home. Doing such chores can help me because, if my wife falls ill, I will be able to cook for her and the children. I just feel I should be prepared for such kind of situations." Carl, patient

#### Sexuality and manhood

Whilst a few men, particularly HIV positive men on ART, managed to renegotiate their masculinity in ways that enabled them to access services, men were generally portrayed as engaging in activities that demonstrated their sexuality and manhood. Men were perceived to be 'sexually unstoppable' and whilst men generally needed to appear in control, this was one area where men could admit to being out of control, a 'weakness' leading men to spend considerable time and money in beer halls.

"After getting drunk, some men lose control and end up fondling any woman they come across, that is a weakness of men. Also, men can easily spend all their earnings on alcohol." Godfrey, patient

Unlike women, it was considered normative for men to have extra-marital relationships. Often these required some level of maintenance, taking much needed money away their wives and children. One patient (Samson) said wives were so used to their husbands' infidelities that they readily forgave them for it.

"Men have this weakness of having extra-marital affairs if they are married, or just having more than one girlfriend. It's unfortunate that women have kind of accepted this weakness of men. So much that, if their husbands do that, they are ready to just forgive him and move on with life." Samson, patient

What Samson's quote underlines is an intrinsic acceptance of local constructions of men's sexuality. Men's need to assert their sexuality and manhood is seen as a 'matter of fact'. Not only does this representation serve to justify men's extra-marital affairs, but it also puts tremendous pressure on men to perform and demonstrate their sexuality/manhood. Men's sexuality, roles and responsibilities are under threat by HIV/AIDS, and the reactions of many men, as the next section will show, serve as barriers to men's uptake of HIV services.

### Barriers to men's HIV services uptake

#### Men's fear of HIV

Many of our research informants spoke of men having a profound fear of HIV, preventing them from timely HIV testing and treatment.

"Men are generally afraid to be known that they are HIV positive. They are shy and they may only come out after they get seriously ill. Some men are afraid that people in the community will laugh at them or look down upon them for being HIV positive." Johnson, patient

Being HIV positive not only compromises a man's sense of masculinity, it is also a sign of a man being unable to control his sexuality. Whilst having multiple sexual partners is a sign of virility, many men reported feeling embarrassed from failing to protect themselves. Fearing they will lose their dignity if found to be HIV positive, many men opt to ignore HIV services, or if they have been tested, hide their status from their wives and do not seek treatment.

"Sometimes they will not even tell the truth to the nurses fearing that everyone might know that they contracted an STD from their sleeping around. I think this is especially true for men. Some men are getting to the extent of dying with these STDs without seeking treatment, they even hide such illnesses to their wives." Spencer, patient

Although it may be counterintuitive for men to report feeling guilty and embarrassed about their extra-marital affairs when having multiple girlfriends is one way to demonstrate their masculinity, it appears that a healthy sexuality is intrinsically linked to a hegemonic masculine sexuality. A man who engages in extra-marital sexual relationships and gets an embarrassing disease like HIV is perceived to have a weak, diseased, compromised, laughable and despicable sexuality - compromising his manhood. Relatedly, admitting to such a tainted sexuality may compromise a man's relationship with his wife. A number of men feared being left alone should their wives learn that they are HIV positive. Also young men feared that their tainted sexuality would limit their chances of being with women and eventually finding a woman who would take of them.

"For the young men who are still single, they also think about whether they would still be able to get married as they fear that their girlfriends may just shun them if they test HIV positive." Sunny, patient

#### Delusion, denial and diversion

Men like to see themselves as all-knowing and dominant. To admit there is something they do not know may imply lack of power and will put them in the position of a 'learner', a subservient and unmanly trait. Grounded in a fear of HIV, many men therefore appear to be in denial regarding the seriousness of HIV and AIDS - preventing them from accessing HIV preventative information and from seeking HIV services. As Spencer suggests, men will actively avoid spaces where AIDS is being discussed.

"I think the reason that is there is men who don't want to come out in the open, they want to hide and they don't have the knowledge. If they hear of a place where AIDS is being discussed, they don't want to go to that place, so men do not have that much knowledge." Spencer, patient

This, coupled with a demonstration of macho fatalistic risk taking and holding on to very rigid, inflexible and insecure positions, means many men continue to be at risk of HIV.

"Men are dying and they don't want to be tested. You hear them saying HIV is like an accident and people cannot stop driving because of accidents, so some men will not change their behavior." Stuart, patient

Men tell themselves many things to justify their behaviours. For example, some see HIV as a disease that only *exceptionally *promiscuous men will contract and therefore not at risk themselves. Illustrating the deep denial of HIV in many men, Godfrey describes the different ways in which he sought to deny that he had contracted HIV.

"I did my own inventory of the women I had had sex with, and I could not even single out one of them as potentially the one who gave me the infection. I really did not believe it. Then I used to feel that I was just unlucky because I knew men who were doing worse things but still going on fit. I got to the extent of even questioning the existence of HIV/AIDS because I thought, if it really existed, why were some very promiscuous men escaping the infection. I also thought even that machine that was used for my HIV tests could have been defective." Godfrey, patient

Whilst Godfrey blamed the HIV testing procedure in his attempt to avoid the reality of AIDS, other men resorted to drinking alcohol. Not only did this contribute to the continued spread of HIV, but also the avoidance of HIV testing and ARV adherence.

"Some men will drink so much that they forget to take their drugs, and even forget that they are patients on ART." Carl, patient

In an attempt to save some dignity, men were reported to blame their wives for bringing HIV into the family. Whilst no men admitted to blaming their wives, this was commonly reported by nurses and female patients.

"Men are stubborn sometimes. They blame the wives for bringing the disease into the home in an attempt to preserve their role as head of household." Bridget, patient

#### Masculinity conflicts with 'patient persona'

Many men were also alarmed by what it means to be an HIV patient. Not only would men have to admit they were ill and own up to their physical limitations, they would also need to enter female spaces and act in ways that conflicted with hegemonic notions of masculinity. To be enrolled onto ART in this context, patients must attend monthly consultations at a hospital. However, due to women's participation in maternal, infant and child health and related visits to the hospital, hospitals are perceived by many men as female spaces, discouraging them from engaging with HIV services in a hospital setting.

"Men view health issues as female issues. Women always go to the hospital from pregnancy and until the children are grown up. So men feel hospitals and health concerns are for women." Michael, patient

Aside from hospitals being perceived as female spaces, there are numerous potential elements of a hospital visit that conflict with masculinity. One challenge that was frequently referred to relates to men's difficulties in showing up for appointments with health staff.

"When you give them the review dates that you want to give him a counselling session, they do not turn up at the time you specify. They just come when they feel like it - on their own free will time. So men are very difficult to deal with." Claudius, nurse

Different reasons were given to why men struggle showing up for review dates. Men are expected to queue up patiently wait outside the HIV clinic. In doing so, a man will not only run the risk of being recognised as HIV positive, but will have to let go of any sense of control of his time and his freedom, having to follow instructions given to him in this biomedical setting and wait patiently like 'one of the women.'

"So men find it hard to just go to the clinic... you can imagine men waiting in these queues here, men do not have a lot of patience to wait in these queues but women are used to coming here and waiting in these queues. A man would really feel belittled to wait in these queues the whole day shoving and jostling with women." Emmanuel, patient

Some men explained that they cannot afford to wait a whole day to be seen by a nurse as this compromised their head-of-house duties. As part of their monthly consultations, ART patients must adhere to a complex and strict treatment regimen where pills must be taken timeously and where certain activities are strongly discouraged. A number of men explained that their work activities made it difficult for them to carry around their medicine and to take it in a timely manner. Furthermore, ART patients are advised to eat healthily, apply creams to sores, quickly respond to potential opportunistic infections, refrain from unprotected sex, smoking and drinking alcohol. These, and many other instructions worry men to such an extent that some fear enrolling onto ART.

"I think some men know that what they are doing is not good for their health, so they fear the nurses advising them to stop a lot of their activities including drinking alcohol." Daya (female), patient

As many men fail to abide by what is expected of an ART patient, at least until they are more comfortable with their new identity as an ART user and have adopted a more ART-friendly masculinity, they are subject to reprimanding from nurses. The frustration experienced by some nurses as a result of men's inability to adhere to treatment results in nurses occasionally giving up on certain male patients as they repeatedly fail to act upon the advice given to them.

It is clear that men face numerous barriers in accessing HIV services. Grounded in a profound fear of HIV/AIDS as well as perceptions and experiences of HIV services, this section has highlighted some of the ways in which men avoid and delay HIV service use in order to protect or demonstrate their masculinity and dignity. With such strong barriers to HIV services, what opportunities are there for men to make use of HIV services?

### Facilitators of men usage of HIV services

Our discussion above indicates that men face very specific challenges in accessing HIV services. However, in the case of our male informants who are now accessing and adhering to ART, it has been possible to overcome these obstacles and construct new masculine identities more in line with the 'patient persona'. In this section, we will highlight some of the pathways through which men can make use of HIV services.

#### Persuasion and need

As men fear the consequences of being HIV positive and how it may adversely impact their representation of their manhood, being persuaded to make use of HIV services from someone whom they trust often served as a strategy to give men the push they needed to make use of HIV services.

"My wife was worried and was always asking about my health. The swellings were not painful to me at all, so she was always asking what sort of disease it was. I told her I was fine but she insisted that I should go back to the hospital and show them those swellings. She would always push for me to go to hospital every day until I felt I should just go to the hospital to honour her wish." Emmanuel, patient

"Usually we will try all means to make the husband come. We sometimes send a message through village health workers or we send it through the husband's best friend to go and talk to him." Claudius, nurse

But not all men could be persuaded and many only got access to HIV services when they were too sick to move and assert their masculinity and ended being brought to the hospital in wheelbarrows.

"Men will only come to us when they are bedridden and brought to us in a wheelbarrow." Collin, nurse

This was the single most frequently mentioned route through which men arrived at hospital and were initiated onto ART. Many of our male participants, who are now living positively and on ART, admitted to also being brought to the hospital in wheel barrows after continuously denying their illness. Having said this, other facilitators of men's HIV service usage were identified and study participants provided us with pointers as to how HIV services and the local environment can help men overcome gender-related obstacles.

#### Gender sensitive HIV management services

Much of what we have discussed so far suggests that the need for gender sensitive HIV services. Men require counselling that challenges the versions of masculinity that prevent them from living positively and on ART. Although these groups were open to both women as well as men, rather than men-only spaces as is the case in other settings [e.g. [33]] our interviews suggested that counselling was often key to helping our male (patient) participants come to terms with their HIV status, demystifying inaccurate perceptions and helping men to step away from hegemonic notions of masculinity - in favour of revised identities that placed key emphasis on previously less important roles, such as being a family man or playing an active role in encouraging other men to make use of available HIV services in a caring and supportive way.

"To be honest with you that counseling opened my eyes to the fact that my HIV status will not necessarily affect my role in the family, rather my family now understands me better. But initially that feeling of being reduced to an HIV sufferer who is good for nothing gripped me, but now I am doing well." Emmanuel, patient

The health clinics in our study area group ART users together into (again mixed gender) support groups to facilitate ART adherence. In the support groups, ART users can negotiate and renegotiate identities that help them both navigate through local struggles and meet the expectations of a 'patient persona'. As outlined by one male patient (Stephen), the support groups allow men to come to terms with their status through acceptance and support from other members as well as access food aid.

"Now we have these support groups that are well known to be a platform for HIV/AIDS sufferers to give each other support; it was never like that before. In the past, nobody would really want to be associated with such a disease, a lot of people now want to get tested so that they can also get some food which is sometimes given to people living with HIV/AIDS." Stephen, patient

It appears that food aid, distributed through support groups for ART users, is highly beneficial to men as it allows men to reconcile the 'patient persona' with their local obligation as breadwinners. This is illustrated by the dramatic increase of men actively seeking HIV testing in the hope that they may be positive and qualify for food aid.

"I had never seen a man coming to be tested when they could still walk until recently when Africare [NGO providing food aid for ART users] came. Now we are seeing more men coming for tests." Lydia, nurse

This sub-section has given a few examples of how HIV services interact with masculinities and provide a platform for men to construct new and more ART-friendly masculinities.

#### Constructing responsible masculinities

In order for men to cope with the stresses of HIV and to make use of HIV services, they often have to undergo a remarkable transformation, breaking free from socialised norms of what it means to be a 'real man' and to live by revised or renewed versions of 'manhood'. This can take a while and many men fail to adhere to ART as they continue to abide to the traits of hegemonic masculinities. However, with time and through self-reflection, as well as the kind of support discussed above, some men are able to construct a new set of representations, another version of masculinity, integrating key traits of hegemonic masculinities with their life circumstances (being HIV positive and on ART). One dominant representation that male patients appeared to adopt is that of being a responsible and valuable citizen. They are socially responsible because they took the difficult step to get tested, have gained a lot of knowledge (through counselling and peer support groups) about HIV/AIDS management and now pass this on to the fearful 'ignorant other.'

"So I have learnt a lot from going to counseling, and now I even encourage fellow men to consider getting tested rather than them suffering in silence, fearing that they may be told that they are HIV positive." Emmanuel, patient

Furthermore, in lieu of representations of AIDS-affected people as unproductive and a burden, unable to care for their families, many of our male informants spoke about how being on ART had enabled them to participate in productive activities and contribute to the long-term of their family, highlighting their social value.

"What makes me look forward to the future is my health which is in a good condition. Also my family. I look forward to building a good future with my family." Nick, patient

Men often spoke about their role in the family and how ART had enabled them to live up this role. In constructing socially responsible masculinities, men also explained how they had stopped drinking alcohol or were no longer engaging in extra-marital affairs - emphasising their family role.

"I have also stopped drinking so that I can concentrate on my treatment [...] I take this programme seriously because my family depends on it." Charles, patient

Their sense of having some kind of control over their health and still being able to fulfil some of the key traits of being a 'real man' has also been a building block for men to resist derogatory stereotypes from their peers. Seeing themselves as responsible and informed and their peers as fearful and 'ignorant', male ART users are able to create a distance from otherwise hurtful attitudes and actions.

"They also use derogatory names and they do it openly, I think they have not yet understood the importance of treating an HIV sufferer as a normal human being. Maybe they don't know that we are just the same." Johnson, patient

This section has outlined some of the opportunities that exist in this context in facilitating men's use of HIV services. Men drew on the facilitators in different ways and at different stages, highlighting the complex transformation process, which many men will have to endure in order to make use of HIV services.

## Discussion

This study responds to Hirsch's [13] call for greater attention to the social processes that result in gender disparities in HIV service uptake. Although a number of studies have alluded to some of the obstacles men are facing in accessing HIV services as a result of social constructions of masculinity [14,15,32,33], this is the first in-depth study, as far as we are aware, that set out to explore explicitly the role of masculinity in influencing African men's use of HIV services.

Our findings suggest that HIV service uptake in this context is deeply intertwined with hegemonic masculinities, intensified by local socio-cultural influences and wider global expectations of HIV/AIDS patients. Hegemonic understandings of masculinity in this cultural context define 'real men' as strong, emotionally independent, tough and fearless. This, coupled with their role as breadwinners, makes it important for many men to reassert their masculinity. In the process of doing so, men in this case study sought to avoid HIV/AIDS services, demonstrating themselves as strong and resilient to illness. They also did so out of fear that their wives would leave them if they found out that they had had extra-marital relationships and brought HIV into the family. Men's fear of HIV led them to either refute the presence of HIV, exercising denial of the risky nature of their behaviour, and refusing to view themselves at risk of contracting the disease. These factors all contributed to men's delay in HIV services uptake.

We also found evidence that hegemonic masculinities compromised men's adherence to HIV treatment. Due to women's maternal health role, hospitals were perceived as predominantly female spaces, and not a place for men to be. Furthermore, therapeutic representations, such as being associated with AIDS, queuing up patiently amongst women in the hospital and taking instructions from nurses (who would e.g. instruct HIV positive men to stop having multiple partners and drinking alcohol and to take an interest in their health, diet and medication regimen), made ART inherently male-unfriendly and therefore difficult for men to adhere to. Whilst these are some of the factors that prevent many men in this context from making use of HIV services, we also identified a number of factors that facilitate men's uptake and adherence to ART. In agreement with another study from Zimbabwe [cf. 4], many men only accessed HIV services when they really needed to, either because their friends and family persuaded them to go and get tested and seek treatment, or because they were so ill that their family brought them to the hospital in wheel barrows. But because many men's first contact with HIV services was generally not voluntary, counselling services that were considerate of the obstacles that men face in sustaining their engagement with HIV services were found to be a key contributing factor to those who were eventually able to engage in ART uptake and adherence. Counselling, support groups and other services (e.g. food aid) available for ART users, all provided men with a platform to re-construct hegemonic masculinities in such a way that they become ART-friendly. In doing so, we observed men to construct masculinities that accentuated them as socially responsible citizens, primarily because they took control over their health and family. But also because they gained a lot of knowledge (through counselling and peer support groups) about HIV/AIDS management, which they then sought to pass on to their 'ignorant' peers. We do however note that, even when men had the opportunity to re-construct their masculinity, they still seemed to focus on more or less universal social requirements of manhood, such as the man's role in sustaining his family. The new ART-friendly versions of masculinity therefore merely sought to reconcile their therapeutic responsibilities with their pre-existing roles as a son, father and husband. This was most clearly illustrated by the impact of food aid on ART users, as food aid gave male patients the opportunity to be *both *an ART patient *and *a breadwinner, notwithstanding the fact they received the food as a hand-out, as opposed to working for it.

Our findings not only suggest there is an urgent need to make HIV services more male friendly, but also provide some useful pointers to how this can be achieved, particularly through the construction of supportive social spaces in which men can re-negotiate more health-enhancing gender identities. In agreement with Colvin and Robins [33], we argue that social support groups (e.g., established by NGOs, local organisations or health clinics) can provide men with the necessary space to renegotiate masculinities that are more aligned to their engagement with HIV services. We therefore recommend strategies that seek to build safe and supportive social spaces for men to collectively discuss and reflect upon the obstacles they are facing. Furthermore, to overcome low and delayed HIV service use, we concur with Bwambale et al. [41] and call for a greater decentralisation of HIV services, recommending that testing and counseling services are incorporated into community and home-based programmes, making HIV services more apparent at a community-level and removing them from spaces that are perceived as predominantly female (e.g., hospitals). Our findings also suggest there is a general need for a more systematic discussion about masculinity at a community-level, acknowledging both the ways in which masculinities favour men and how masculinities serve as a barrier for men's access to health services. We therefore recommend the support of school-based and community-driven initiatives that can facilitate such discussion. Barker and Ricardo [32] report on eight promising programmes in sub-Saharan Africa that already seek to promote gender-equity by engaging young men and they highlight some useful facilitating factors in achieving this: i) the endorsement of community leaders who publicly support the deconstruction of health-damaging masculinities, ii) the use of role models, iii) mobilizing peer-based groups and safe social spaces, iv) committed and sustained funding, v) local and national alliances and vi) use of visual media and fun activities to engage young men.

Our findings and recommendations should be read against the fact that although men and boys are complicit with, subject to or resistant to hegemonic versions of masculinity, they can adopt different parallel positions in different contexts and for different audiences [42]. Although we strived to do the study in a relatively homogenous population of working class, poorly educated, rural Shona people, we also acknowledge that hegemonic masculinities vary from one context to another, influenced by class, educational attainment and ethnicity [12,43].

Although this paper set out to explore obstacles and barriers to men's use of HIV services, the findings also offer potential explanations - which deserve further exploration - to why women fare better in making use of HIV services. Numerous references were made to hospitals as female spaces, which, coupled with an increasing number of pregnant women getting tested for HIV to prevent mother-to-child HIV transmission [44], helps women access HIV services relatively early on. Furthermore, if the 'patient persona' is characterised by what locally may be perceived as feminine traits, then what are simultaneously obstacles to men may well be facilitators to women. Having said this, women's continued marginalisation, as well as many social and poverty-related factors still prevent many women from accessing and adhering to HIV treatment in Zimbabwe [35,36,38,40].

## Conclusion

A clear and explicit version of hegemonic masculinity emerged across our interviews. A 'real man' is strong, in control, disease free, sexually promiscuous and the breadwinner of his family. Men are not only expected to abide by such representations, but also play an active role in constructing such representations by continually demonstrating their manhood. Such understandings and demonstrations of masculinity conflict sharply with the ART 'patient persona', which requires men to be concerned about their health and regularly go to the hospital - a space many men see as a 'female space' - take instructions from nurses and refrain from unprotected and extra-marital sex and alcohol. However, it was equally clear that not all men subscribed to it. Once men had been counselled and had the opportunity to reflect upon the impact of ART on their productivity and social value, it was possible for men who had initially subscribed to hegemonic masculinities to construct new and more ART-friendly versions of masculinity - enabling them to adhere to their treatment. We conclude that men's use of HIV services depend on (i) the social constructions of masculinity that characterise a context, (ii) the openness and ability of the context *and *men living within it to discuss and deconstruct hegemonic masculinities and (iii) men's on-going negotiation between the 'patient persona' and social constructions of masculinity, helping them construct ART-friendly masculinities.

## Competing interests

The authors declare that they have no competing interests.

## Authors' contributions

MS performed the data analysis and drafted the manuscript. CC designed the study and finalised the manuscript. CM and ZM conducted the interviews and prepared the data for analysis. CN and SG coordinated the study, participated in the design of the study and commented on the manuscript. All authors read and approved the final manuscript.

## Endnote

^i ^HIV services in this paper refer to HIV testing and antiretroviral therapy
